# Tubercular serpiginous choroiditis

**DOI:** 10.1186/s12348-022-00312-3

**Published:** 2022-11-09

**Authors:** Reema Bansal, Vishali Gupta

**Affiliations:** Advanced Eye Centre, Post Graduate Institute of Medical Education and Research, Chandigarh, 160012 India

**Keywords:** Immune mechanisms, Pathogenesis, Serpiginous choroiditis, Serpiginous-like choroiditis, Tubercular

## Abstract

Tubercular association with serpiginous choroiditis, also called ‘serpiginous-like choroiditis’ or ‘multifocal serpiginoid choroiditis’ (MSC) is reported from world over, especially from endemic countries. Though the exact mechanism is not yet clear, a direct or indirect infectious trigger by *Mycobacterium tuberculosis* (MTB) is believed to cause choroiditis.

The link of immune mechanisms with ocular inflammation caused by MTB is emerging, and has been supported by both experimental and human data. The molecular and histopathological findings of tubercular serpiginous-like choroiditis have been demonstrated in clinicopathological reports, as well as in animal models. Young to middle-aged healthy males are more frequently affected. The choroiditis lesions of tubercular serpiginous-like choroiditis evolve as multifocal lesions, affecting the retinal periphery as well as posterior pole. They begin as discrete lesions, and spread in a serpiginoid pattern to become confluent. Fundus imaging including autofluorescence is extremely helpful in monitoring patients for response to therapy. Its diagnosis is essentially clinical. Corroborative evidence is obtained by a positive tuberculin skin test, or a positive QuantiFERON-TB Gold (Cellestis, Carnegie, Victoria, Australia) test, and/or radiological (chest X-ray or chest CT scan) evidence of TB elsewhere in the body. Systemic corticosteroids are the mainstay of therapy to control active inflammation, while ATT helps to reduce recurrence of inflammatory attacks. Immunosuppressive agents are indicated in cases with relentless progression, paradoxical worsening, or recurrent choroiditis.

Serpiginous choroiditis (SC), previously known as geographic choroidopathy [1], or helicoid peripapillary chorioretinal degeneration [2] is a chronic, bilateral, recurrent inflammation of the choroid and choriocapillaris, [3, 4]. The retinal pigment epithelium (RPE) and retina get secondarily affected. With no definitive cause known often, an autoimmune mechanism has been postulated to cause SC. Though not common, SC has been associated with a few known systemic diseases, such as sarcoidosis [5, 6], Crohn’s disease [7], polyarteritis nodosa [8], systemic lupus erythematosus [9], non-Hodgkin’s lymphoma [10], and Celiac disease [11].

Among the infectious etiologies causing choroiditis mimicking SC, tuberculosis (TB) is the commonest [12–27]. Others include toxoplasmosis [28], viral infections [29, 30], fungal infection [31], syphilis [32] and others [33]. Recently, SC has been reported following SARS-CoV-2 infection [34–36].

Tubercular association with this variant was coined the term ‘serpiginous-like choroiditis’ (SLC) in 2003 [13], and subsequently ‘multifocal serpiginoid choroiditis’ (MSC) in 2012 [14], to differentiate this variant from the classic SC. Though the diagnosis of both is essentially clinical, the two have several phenotypic differences.

## Epidemiology and demography

Tubercular SLC (TBSLC) is considered to be affecting people living in endemic areas [15, 16, 19, 37]. However, it is recently being increasingly recognized in non-endemic areas too [23, 25]. Among the three phenotypes of tubercular choroiditis, TB SLC is the commonest, followed by focal/multifocal choroiditis and tuberculoma [38]. Men are more frequently affected than females, in young to middle-age group.

## Pathogenesis: immunological aspects

The first association of TB with SC was given by Hutchinson in 1900 [39]. While various autoimmune mechanisms have been postulated for classic SC [40–42], a direct or indirect infectious trigger by *Mycobacterium tuberculosis* (MTB) has been implicated in TBSLC, by demonstrating the presence of MTB in the vitreous, aqueous, retinal pigment epithelium (RPE) or choroid [13, 19, 20, 43]. More commonly, although presumptive, presence of latent MTB infection or a systemic, extraocular focus of MTB infection provide etiological association of MTB with SLC [14–17].

The immune mechanism of tuberculous infection within the human eye has a very limited information. The role of autoimmunity and autoinflammation is emerging in infectious or undifferentiated uveitis, by demonstrating both cellular and humoral responses to several retinal antigens [44–47]. Forrester et al. described the link of immune mechanisms with ocular inflammation caused by MTB [44]. The MTB targets macrophages and dendritic cells (DC) during the initial infection of the lung. While the MTB either kills them or gets killed by them, it escapes the other myeloid cells and resides in them. These cells latently infected with MTB recirculate out of the granulomas and travel to extrapulmonary sites (lymph nodes, kidney, muscle, meninges and uvea). It resides in these tissues and may get reactivated any time later. Caseation necrosis of the tissue occurs when MTB thrives, causing local infection. In immunocompetent individuals, when the MTB is contained and gets reactivated, it elicits an intense host immune response, causing immune-mediated inflammation and damage. The dead cells release MTB antigen that stimulates innate immune cells to react with autoreactive T cells, leading to a secondary autoimmune response [47].

### Experimental data supporting immune mechanisms

In a rabbit model of experimental autoimmune uveitis (EAU), granulomatous inflammation was induced in the uveal tissue (iris, ciliary body and choroid) by injecting dead MTB bacilli into the common carotid artery [48]. As the dead bacilli could not cross the blood-retinal-barrier (BRB), the inflammation did not involve the retina.

The choriocapillaris and RPE are the key sites of inflammation in TBSLC. In a mouse model, the viable MTB could induce inflammasome activation in the RPE through its two constituents, Early Secreted Antigenic Target-6 (ESAT-6) and mycobacterial RNA (double stranded) [49]. Both ESAT-6 and mycobacterial RNA represent microbial viability. Even when ESAT-6 was injected in subretinal space, the inflammasome was activated in mice. The MTB protein ESAT-6 is one of the key virulence factors of MTB. It is also a potent activator of NLRP3 (NOD-like receptor family, pyrin domain containing 3) inflammasome. The study demonstrated the expression of NLRP3 in human TBU specimens, and ability of both ESAT-6 and RNA components from MTB (even in the absence of complete MTB bacilli) to activate the inflammasome in iris, choroid, retina and RPE in human TBU, suggesting an innate immune response.

Immunization with retinal antigens (combined with Complete Freunds Adjuvant containing heat-killed MTB) induces an autoimmune response (T cell mediated) in the retina and other tissues, which forms the basis of EAU [50]. It is likely that a similar mechanism causes uveitis in human eyes, by the MTB antigens released from the dead or live bacilli present within the eye or at an extraocular site, which cause priming of autoreactive T cells.

In another animal model, evidence of intraocular inflammation was seen after systemic and local priming of the rat with injection (subcutaneous followed by intravitreal a week later) of mycobacterial antigen (Mtb H37Ra). This may be extrapolated to the mechanism of uveitis seen in humans with corroborative (immunological and/or radiological) evidence of past MTB infection [51, 52].

### Human data supporting immune mechanisms

Tagirasa et al. identified autoreactive T cells in eyes with tubercular uveitis (TBU) [47]. The clinical phenotypes of TBU in their series included retinal vasculitis, MSC (or SLC), focal choroiditis and intermediate uveitis. They analysed the intraocular T cell response in vitreous fluid, and found it to be highly pro-inflammatory, involving effector and central memory T cells. Regarding the cellular source of these cytokines, they found CD4^+^ cells to be the predominant phenotype in all eyes with TBU. Further, they detected polyfunctional cytokine responses to MTB-secreted ESAT-6 peptides, suggesting that the T cell response within the vitreous was directed against active MTB infection. Also, absence of this cytokine response to ESAT-6 in the corresponding blood samples of TBU patients indicated the localized nature of MTB-specific response, being restricted to the eye. Breakdown of BRB, caused by MTB-induced inflammation, allows entry of peripheral autoreactive cells into the eye. The cognate self-antigens, which are significantly more abundant than MTB antigens in the eye, activate the peripheral autoreactive cells to proliferate within the eye. A prolonged or recurrent intraocular inflammation is probably attributed to relative resistance of autoreactive T cells to antigen-induced cell death. This study provided a definitive evidence of both direct (intraocular T cell response to ESAT-6, the mycobacterial antigen) and an indirect (cytokine production by intraocular T cells to retinal crude extract or RCE) response in eyes with TBU, including TBSLC. However, it is not known whether the T cells reacting to two different antigens (mycobacterial antigen and RCE antigen) represent two different populations within the eye, indicating a cross-reactivity. Also, whether the autoreactivity of T cells is an epiphenomenon needs to be explored.

Subsequently, Basu et al. critically reviewed two main mechanisms causing inflammation in the eye due to TB: a direct MTB-driven inflammation, caused by live/replicating MTB within the eye, and an indirect inflammatory response (immune mediated) to non-viable MTB (or its components) within the eye [53]. A combination of both these mechanisms is likely to play a role in various phenotypic presentations of TBU.

An increase in the proinflammatory cytokines in intraocular fluid (vitreous/aqueous humour) of TBU patients has also been reported by a few studies [54–56]. Sharma et al. studied the cellular composition and local cytokine response in vitreous of TBU patients (intermediate or posterior uveitis) [56]. The major fraction of infiltrating T cells included CD3+, CD4+ and CD8+ T cells. These T lymphocytes were found to be recently activated, and released IL-17A and IFN-γ (proinflammatory cytokines) in the vitreous. The higher level of these cytokines in the vitreous than in peripheral blood of these patients suggested that the antigenic trigger was local rather than systemic in origin. The authors also studied the association of Toll-like receptors (TLRs) with TBU, and found a hypo-responsiveness of activated CD4+ T cells in the vitreous to TLR9 agonist ODN 2216 FITC.

In MSC, the serum cytokine levels were studied at baseline and over a longitudinal follow up in patients who received oral corticosteroids and anti-tubercular therapy (ATT) [57]. Of 12 patients, four developed paradoxical worsening (PW) of choroiditis lesions. As compared to patients with complete healing of lesions (eight patients), those with PW (four patients) had a higher IL-10 at baseline, significantly raised IFN-γ levels at 1 week, and significantly raised TNF-α levels at 3 weeks in the serum, during the time of occurrence of PW. The two groups did not differ in TGF-ß levels. This indicated that the pro-inflammatory cytokines increased in the serum during the event of PW due to a higher bacillary load. These cytokines could serve as markers in predicting the treatment outcome with ATT and steroids in patients with TBMSC. A higher mycobacterial antigenic load at baseline may predispose TBMSC patients to develop PW, who demonstrate an increased immune response after initiating ATT.

## Pathogenesis: molecular and histopathological aspects

The molecular and histopathological findings of TBSLC have been demonstrated in clinicopathological reports, as well as in animal models [19, 20, 43, 48, 49, 58, 59]. Bansal et al. used three different molecular techniques (multitargeted PCR for MTB assay, Gene Xpert MTB/RIF assay, and a line probe assay) and detected MTB genome in vitreous fluid of eyes with TBSLC [19]. This study was the first to report rifampicin resistance in eyes with TBSLC.

In an enucleated eye with panuveitis of unknown cause, histological sections of the globe revealed inflammation (granulomatous) of the uveal tissue and retina [20]. The RPE showed necrosis, with acid-fast bacilli sequestered within the RPE cells, suggesting that RPE was the preferred site for localization of MTB in eyes with panuveitis or tubercular choroiditis (TBSLC). These sequestered bacilli in the RPE represent the phagocytosed bacilli (as in macrophages in pulmonary TB), which thrive by avoiding phagolysosome fusion. Their reactivation in the RPE probably results in recurrence of TB SLC.

Kawali et al. reported clinicopathologic features of TB SLC in a 28-year-old male. Following progression of macular choroiditis upon initiating ATT, the patient underwent a vitreous and chorioretinal biopsy [43]. The biopsy revealed caseous necrosis and granulomatous inflammation of the inner choroid, with degeneration of RPE and disruption of photoreceptors. This case probably represented the histopathologic findings of PW (previously unknown) in TBSLC.

Experimental choroiditis (besides other types of uveitis) developed in rabbit eyes following injection of living tubercle bacilli into the common carotid artery, as reported by Finoff in 1924 [48]. While majority of animals developed some form of systemic TB, all 46 animals developed ocular lesions, on the side injected. Clinically, the choroidal lesions manifested as ill-defined, oval, yellow patches, and healed to the atrophic stage with pigmentary changes. Histologically, the choroid was infiltrated with epithelioid cells, with clumps of bacilli seen centrally, without involvement of RPE in the early stages. Subsequently, with RPE degeneration and proliferation, pigment granules invaded retina. Later, the retina became completely atrophic over the lesion area, with caseation in the centre. This study proved the hematogenous origin of ocular TB, beginning in the iris, then cornea, followed by conjunctiva and lids, and finally the choroid.

In mouse RPE cells, NLRP3-dependent caspase-1 activation and inflammasome priming was induced by two constituents (ESAT-6 and double stranded mycobacterial RNA) of viable MTB [49]. In tissue sections of human ocular TB, both retina and RPE demonstrated NLRP3 staining. This study reported immunolocalization of NLRP3 and double stranded mycobacterial RNA in human ocular TB sections.

## Clinical features

Young to middle-aged healthy males are more frequently affected. The choroiditis lesions of SLC or MSC evolve as multifocal lesions, affecting the retinal periphery as well as posterior pole. They begin as discrete lesions, and spread in a serpiginoid pattern to become confluent. Healing of the lesions starts from the centre. Inflammation of the vitreous is common, and anterior segment is seldom involved. Occasionally, placoid and solitary lesions may be seen. The affected eyes usually have both active as well as healed lesions. Healed lesions show scarring and RPE atrophy with prominent underlying choroidal vessels. Hyperpigmentation of the scars is a feature of long duration of the disease, with or without subretinal fibrosis. Final visual acuity is generally preserved as the fovea escapes extensive pigmentation and scarring.

Retinal vasculitis, cystoid macular edema and optic disc edema are some of the rare associations, which may co-exist in the same eye with MSC [14, 22].

In contrast, SC affects elderly patients, and the choroiditis lesion in SC (autoimmune) is large, solitary and juxtapapillary. Vitritis is minimal or none. Tubercular MFC frequently shows multifocal, confluent lesions (that are non-contiguous to optic disc) in posterior pole as well as periphery, with vitreous cells.

## Fundus imaging

**Fig. 1:**
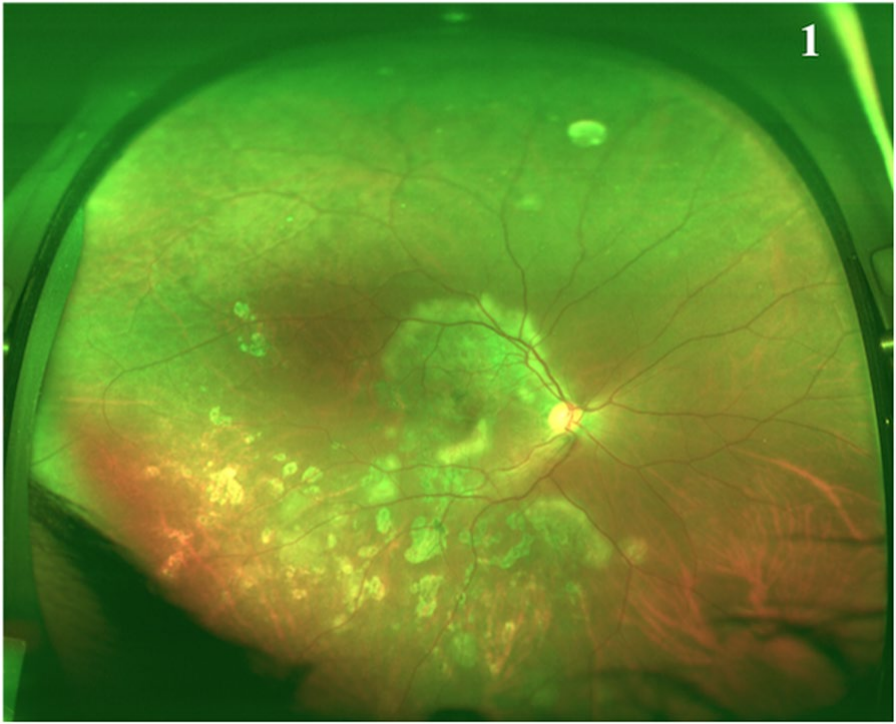
Ultra widefield fundus photograph of an eye with tubercular serpiginouslike choroiditis

Fundus imaging at baseline is important to document the extent of involvement, and helps to monitor the response to therapy at follow up. The outcome of MSC lesions (resolution, progression or recurrence) is best seen through digital fundus photography. Wide-field imaging (Fig. 1) is evolving as the preferred modality for capturing peripheral lesions of MSC [60, 61].

**Fig. 2:**
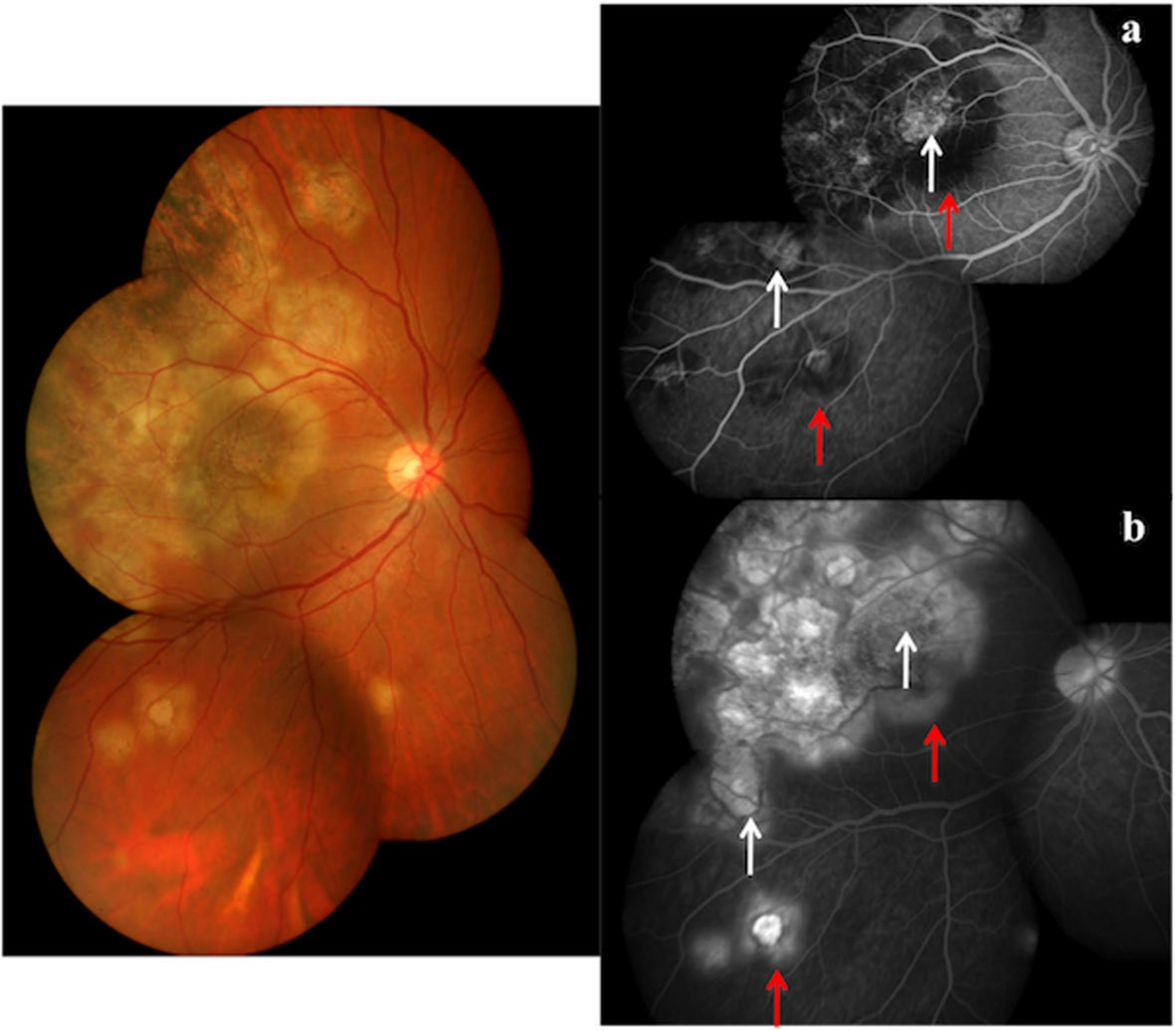
Color fundus photograph showing active lesions of TB SLC (left panel). Fluorescein angiography in early phase **a** showing transmission hyperfluorescence in the centre of lesions (white arrows) corresponding to healed lesions and hypofluorescence along the border of lesions (red arrows) corresponding to active lesions. The late phase **b** shows transmission hyperfluorescence persisting in the centre of lesions (white arrows) corresponding to healed lesions and hyperfluorescence along the border of lesions (red arrows) corresponding to active lesions

**Fig. 3:**
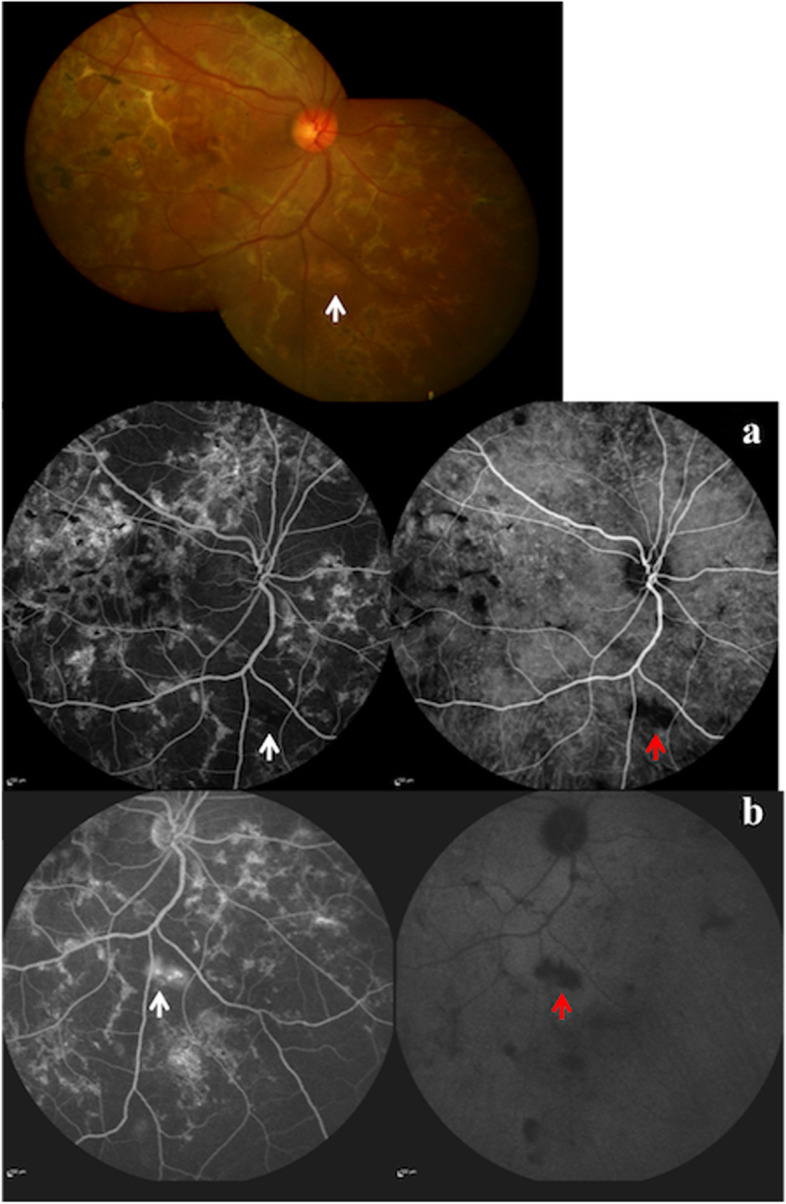
Color fundus photograph showing predominantly healed lesions of TB SLC (top panel) and a few subtle active lesions (white arrow). Combined fluorescein and indocyanine green angiography in early phase **a** shows hypofluorescence (white arrow) and hypocyanescence (red arrow) along the active lesion. Late phase **b** shows hyperfluorescence (white arrow) and persisting hypocyanescence (red arrow)

An initial (Fig. 2a) hypofluorescence and late (Fig. 2b) hyperfluorescence (with ill-defined border) on fundus fluorescein angiography (FA) suggests active lesions. Healed lesions show transmission defects due to RPE loss, appear well demarcated with staining of sharp edges [62, 63]. Hypocyanescence in early (Fig. 3a) as well as intermediate (Fig. 3b) phases is a feature of active lesions on indocyanine green angiography (ICGA), which persists as hypocyanescence in late phase, or becomes isocyanescence [62, 64]. The ICG is superior to fundus examination and FA in identifying subclinical active lesions [65, 66].

**Fig. 4:**
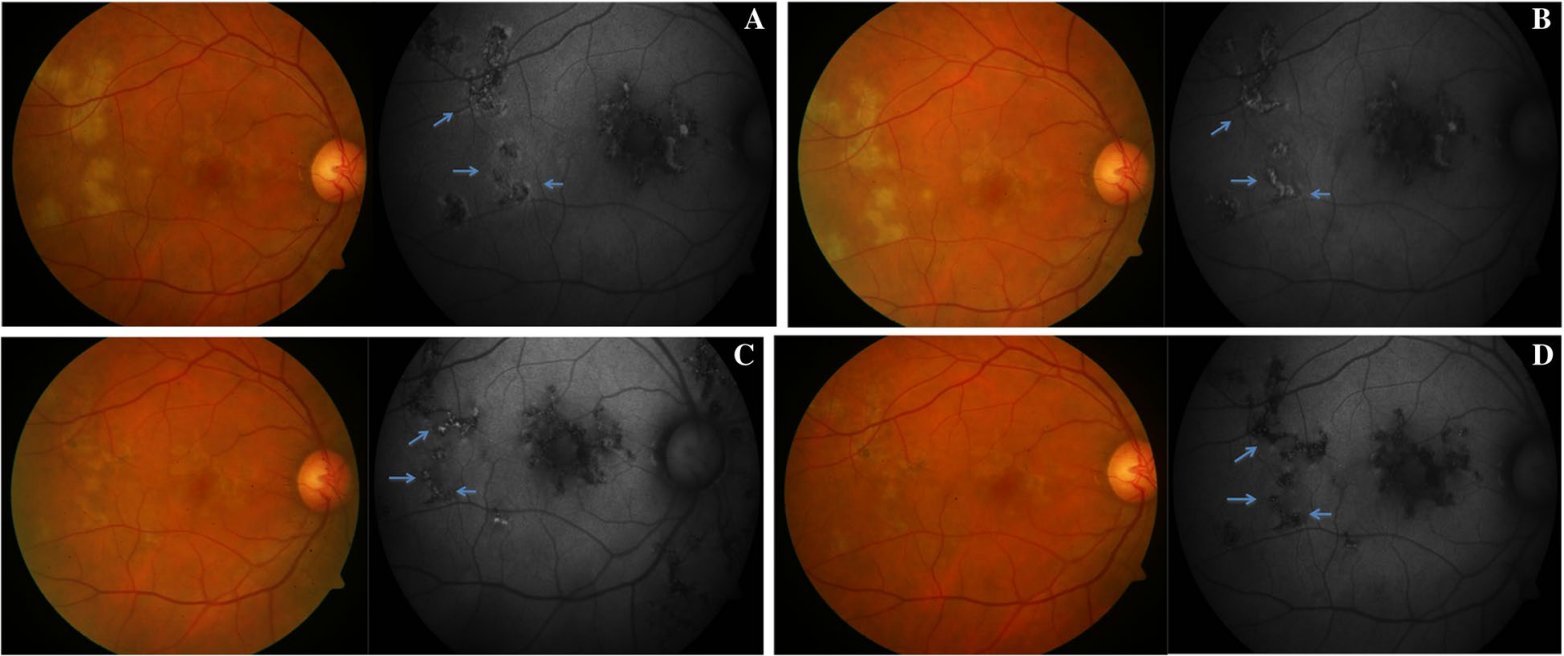
Color fundus photograph (left panel) showing active lesions of TBSLC and fundus autofluorescence (right panel) showing stage I (acute) lesions (blue arrows), characterized by an ill-defined, diffuse halo of hyper autofluorescence along the active lesion (**a**). In stage II (subacute), a thin hypoautofluorescent rim (blue arrows) appears at the border of the lesion as it begins to heal, and the central hyperautofluorescence becomes prominent (**b**). In stage III, as the central hyper autofluorescence decreases and peripheral hypoautofluorescence increases (blue arrows), the lesion appears stippled (**c**). In stage IV, with complete healing of the lesion over several months, there is generalized hypo autofluorescence (blue arrows) (**d**)

Fundus autofluorescence (FAF), based on the property of fluorescence of lipofuscin, is extremely crucial in MSC [18, 21]. It has the advantage of being a non-invasive, and a quick modality, when compared to conventional FA and ICGA. It is much more reliable (than clinical examination, FA or ICGA) when active lesions are just evolving and subtle, or when treated lesions are healing. Typically, a new active lesion evolves through four stages of FAF in tubercular MSC as it heals completely. In stage I (acute), an ill-defined, diffuse halo of hyper autofluorescence corresponds to the active lesion (Fig. 4a). In stage II (subacute), a thin hypoautofluorescent rim appears at the border of the lesion as it begins to heal. The central hyperautofluorescence becomes prominent (Fig. 4b). In stage III, as the central hyper autofluorescence decreases and peripheral hypoautofluorescence increases, the lesion appears stippled (Fig. 4c). In stage IV, with complete healing of the lesion over several months, there is generalized hypo autofluorescence, which becomes intense as scarring sets in with time (Fig. 4d).

These FAF changes correlate with changes in outer retinal layers (RPE, photoreceptors, external limiting membrane and outer nuclear layer) as reflected on high-resolution spectral domain optical coherence tomography (SD-OCT) [21]. Enhanced-depth imaging (EDI) OCT reveals diffuse choroidal thickening in acute stages of MSC [67]. On healing, the choroid below the MSC lesions becomes atrophic, while the choroid underneath the unaffected retina returns to normal thickness and contour. The OCT also aids in detecting associated complications (choroidal neovascular membrane, cystoid macular edema, macular scar, epiretinal membrane, etc.)

## Investigations

The diagnosis of tubercular SLC is often clinical. Additionally, a history of living in TB-endemic areas, or contact with a person with TB, aids in strengthening the diagnosis. Corroborative evidence is obtained by a positive tuberculin skin test, or a positive QuantiFERON-TB Gold (Cellestis, Carnegie, Victoria, Australia) test, and/or radiological (chest X-ray or chest CT scan) evidence of TB elsewhere in the body [15–17, 68–70]. Though an expensive test, PET-CT may be ordered to detect a systemic focus of TB [71–73]. Detection of tubercular DNA in intraocular fluids by PCR provides a definitive diagnosis of tubercular etiology in MSC [19, 20].

## Treatment

Systemic corticosteroids are the mainstay of therapy to control active inflammation, while ATT helps to reduce recurrence of inflammatory attacks [13–15, 19]. In patients who develop paradoxical worsening of inflammation a few weeks following the initiation of ATT, an increase in the dose of systemic corticosteroids controls inflammation [26]. Immunosuppressive agents are used in cases with relentless progression, or recurrent choroiditis [19]. Intravitreal steroids (sustained release dexamethasone implant) are an option for unilateral cases or to avoid side effects of systemic steroids, or as an adjunct with conventional oral corticosteroids and ATT [74].

## Data Availability

Not applicable.
